# Dissecting SOX9 dynamics reveals its differential regulation in osteoarthritis

**DOI:** 10.1002/jcp.31443

**Published:** 2024-09-29

**Authors:** Kannan Govindaraj, Sakshi Kannan, Rodrigo Coutinho de Almeida, Lucas Jansen Klomp, Marcel Karperien, Ingrid Meulenbelt, Janine N. Post

**Affiliations:** ^1^ Department of Developmental Bioengineering, Faculty of Science and Technology, Technical Medical Center University of Twente Enschede The Netherlands; ^2^ Department of Biomedical Data Sciences, Section Molecular Epidemiology Leiden University Medical Center Leiden The Netherlands; ^3^ Department of Applied Mathematics University of Twente Enschede The Netherlands; ^4^ Present address: Science coordinator (Telespazio) for the European Space Agency Leiden The Netherlands

**Keywords:** cartilage, Cellular heterogeneity, DNA localization, FRAP, Protein dynamics, Transcription factor dynamics

## Abstract

The transcription factor SOX9 is integral to tissue homeostasis and is implicated in skeletal malformation, campomelic dysplasia, and osteoarthritis (OA). Despite extensive research, the complete regulatory landscape of SOX9 transcriptional activity, interconnected with signaling pathways (TGFβ, WNT, BMP, IHH, NFκB, and HIF), remains challenging to decipher. This study focuses on elucidating SOX9 signaling in OA pathology using Fluorescence Recovery After Photobleaching (FRAP) to assess SOX9 activity directly in live human primary chondrocytes (hPCs). Single cell FRAP data revealed two distinct subpopulations with differential SOX9 dynamics, showing varied distribution between healthy and OA hPCs. Moreover, inherently elevated SOX9‐DNA binding was observed in healthy hPCs compared to preserved and OA counterparts. Anabolic factors (BMP7 and GREM1) and catabolic inhibitors (DKK1 and FRZb) were found to modulate SOX9 transcriptional activity in OA‐hPCs. These findings provide valuable insights into the intricate regulation of SOX9 signaling in OA, suggesting potential therapeutic avenues for modulating SOX9 activity in diseased states.

## 1 INTRODUCTION

The transcription factor SRY‐Box Transcription Factor 9 (SOX9) is a DNA‐binding protein and a key player during embryonic development and skeletal formation (Kogata et al., 2018). In the process of cartilage and bone formation, SOX9 plays an important role in mesenchymal stem cell condensation and chondrocyte differentiation (Jo et al., 2014). Haploinsufficiency or heterozygous mutations within and around the SOX9 genetic locus causes skeletal malformation syndrome, and campomelic dysplasia (CD). Children born with CD are known to have shortening and bending of long bones, cleft palate, and other skeletal defects due to abnormal cartilage development (Csukasi et al., 2019). In addition, impaired SOX9 function is often implicated in diseases such as osteoarthritis (OA), fibrosis, micrognathia, dwarfism, cancer, etc. (Jo et al., 2014; Lefebvre & Dvir‐Ginzberg, 2017).

SOX9 plays a well‐established role in OA pathophysiology (Lefebvre & Dvir‐Ginzberg, 2017). OA is a multifactorial, degenerative joint disease and its pathophysiology at the molecular level is yet unknown. Heterogenic chondrocytes are the only cell type present in the cartilage tissue (Dowthwaite et al., 2004). During OA, chondrocytes undergo hypertrophic differentiation and extracellular matrix (ECM) in the cartilage tissue is degraded and its repair and regeneration capacity are limited (Coaccioli et al., 2022). ECM is composed of collagens (type II, IX, and XI), aggrecan, proteoglycans, and glycosaminoglycans (Chen et al., 2014). During the progression of OA, the expression of these ECM genes is downregulated. SOX9 regulates the expression of many ECM genes, such as collagen II (COL2A) and aggrecan (ACAN) in chondrocytes (Wang et al., 2003). SOX9 activity is high in healthy chondrocytes and downregulated during hypertrophic differentiation (Chen et al., 2014; Lefebvre & Dvir‐Ginzberg, 2017; Wang et al., 2003).

Several signaling pathways, including the BMP7, WNT3A, IL1, and TGFβ pathways, have been studied extensively to understand SOX9 transcriptional regulation and OA pathology (Yao et al., 2023). BMP7 is an anabolic factor in cartilage homeostasis and induces expression of ECM proteins, including ACAN and COL2A, and downregulates expression of catabolic enzymes, such as aggrecanase and matrix metalloproteinases (MMPs) (Badlani et al., 2009). WNT signaling is essential for chondrocyte differentiation and endochondral ossification. However, WNT activation is a driving factor of hypertrophic differentiation (Dong et al., 2006). Pro‐inflammatory cytokine IL1β is one of the early catabolic factors expressed in OA and upregulates ECM degrading enzymes (Daheshia & Yao, 2008). Both WNT and IL1β signaling plays a key role in OA pathophysiology. In each of these signaling pathways, SOX9 is a direct or indirect target for regulation (Neefjes et al., 2020). The precise mechanism and the interplay between these signaling pathways regulating SOX9 activity are not yet fully understood (Lefebvre & Dvir‐Ginzberg, 2017).

In this study, using FRAP, we have investigated SOX9 dynamics in healthy, preserved, and OA human primary articular chondrocytes (hPCs) in response to extracellular stimulation. We show that at least two populations of cells are present in healthy, preserved, and OA hPCs, and they respond differently to external stimulation. Higher spatiotemporal resolution of our FRAP data has also captured distinct nuclear localization patterns and dynamics of SOX9‐mGFP among healthy, preserved, and OA hPCs. Moreover, we have correlated SOX9 target gene expression with its dynamics.

## 2 MATERIALS AND METHODS

### 2.1 Cell culture and transfection

Healthy human primary articular chondrocytes (hPCs) without known OA disease were purchased from Articular Engineering LLC, Illinois, USA. Preserved and OA hPCs were isolated from the knee joint of patients undergoing joint replacement surgery and the donor details are given in Table S1. The collection and use of human cartilage was approved by the local hospital ethical committee (Medisch Ethische Toetsingscommissie (METC)) and for all samples informed written consent was obtained. Preserved chondrocytes were isolated from visibly undamaged regions of an OA joint. hPCs were cultured in chondrocyte proliferation media containing DMEM (Invitrogen) supplemented with 10% FBS (F7524, Sigma), 20 mM ascorbic acid 2 phosphate (A8960, Sigma), and non‐essential amino acids, at 37⁰C with 5% CO_2_. hPCs were expanded and used within 4 passages. Lipofectamine LTX with Plus Reagent (Life Technologies) was used for the transfection of SOX9‐mGFP (Govindaraj et al., 2019), and the manufacturer's protocol was followed.

### 2.2 Histology staining

Tissues were fixed in 4% w/v paraformaldehyde/10% neutral buffered formalin, decalcified in 12.5% w/v EDTA pH 8.0, and embedded in paraffin. After embedding 5 µm sections were mounted onto a glass slide and were deparaffinized by incubating in xylene (4055‐9005, Klinipath) for 5 min (2x) and rinsed with 100% ethanol (0050.41.210.5, Assink Chemie). Tissue sections were rehydrated by incubating the slides in an ethanol series in the following order 100%, 100%, 96%, 90%, 80%, and 70%, 2 min per series. Following rehydration, slides were washed with demi water for 1 min and incubated in hematoxylin solution (GHS332‐1L, Sigma) for 8‐10 min and the excess stain was washed away with tap water. The slide was destained by being 3x dipped in 0.25% acid ethanol (297.5 ml dH_2_O, 2.5 ml concentrated HCl, and 700 ml 100% ethanol) and rinsed with tap water for 15 min. The tissue section was dehydrated by incubating the slides for 2 min in an ethanol series with the following order 70%, 80%, 90%, and 96% and stained with eosin (HT110132‐1L, Sigma) for 15 s and the excess stain was removed by dipping the slides in 100% ethanol. Finally, the slides were incubated in xylene for 5 min (2x) and immediately mounted with GLC mounting medium (1408, Sakura) and air dried. Stained tissue sections were imaged using a Nanozoomer with a 60x objective (Hamamatsu).

### 2.3 Imaging buffer

Imaging was performed in Tyrode's buffer with freshly added 20 mM glucose (GIBCO) and 0.1% BSA (Sigma) (Lidke et al., 2004). Tyrode's buffer is composed of 135 mM NaCl (Sigma), 10 mM KCl (Sigma), 0.4 mM MgCl_2_ (Sigma), 1 mM CaCl_2_ (Sigma), 10 mM HEPES (Acros organics), pH adjusted to 7.2, filter sterilized and stored at ‐20.

### 2.4 Cytokines and inhibitors treatments

Before FRAP measurements, hPCs were treated with either cytokines or cytokines with inhibitors or inhibitors alone in the imaging buffer at concentrations indicated below. For cytokines or inhibitor treatments: hPCs were kept in the imaging buffer containing respective cytokines/inhibitors for 20 min (unless otherwise stated) before FRAP measurements. For cytokines with inhibitors treatments: hPCs were first treated with the inhibitors for 20 min and then cytokines were added to the imaging buffer incubated for another 20 min before FRAP measurements. Only for BMP7 treatment, incubation time was increased to 60 min before FRAP measurements. Control (no treatment), BMP7 (100 ng/ml; 354‐BP, R&D systems), IL1β (10 ng/ml; 200‐01B, Peprotech), WNT3A (10 ng/ml; 5036‐WN, R&D systems), GREM1 (100 ng/ml; 5190‐GR, R&D systems), DKK1 (10 ng/ml; 5439‐DK, R&D systems), FRZB (10 ng/ml; 7584‐SF, R&D systems), IL1Ra (10 ng/ml; SRP3084, Sigma), 1400 W (100 μM; 81520, Cayman Chemicals).

### 2.5 Fluorescence recovery after photobleaching

hPCs (40,000 cells/well) were plated on glass cover slips (12 mm, Ø) in a 24‐well plate and transiently transfected with SOX9‐mGFP a day before FRAP experiments. Cells were maintained in an imaging buffer, with or without cytokines, cytokines with inhibitors, or inhibitors during FRAP measurements. The FRAP measurements were performed using a Nikon A1 laser scanning confocal microscope (Nikon) with 60X/1.2 NA water immersion objective, 488 nm Argon laser at 0.35% (0.12 μW, at the objective) laser power for SOX9‐mGFP. The temperature was maintained at 37°C with an OkaLab temperature controller. For FRAP, a frame size of 256×256 pixels covering the whole nucleus was scanned at 4 frames/sec for 60 s post‐bleach. The pixel size was 0.12 μm with a zoom size of 7.09. A representative circular region of 2.9 μm diameter was bleached with one iteration (60 ms) of 50% (34.3 μW) laser power. Twenty‐five pre‐bleach images were taken, and the last 10 pre‐bleach fluorescence intensity values were averaged to normalize the post‐bleach fluorescence recovery curve. We performed FRAP experiments on at least 40 cells per condition. Since our data was not showing normal distribution, to assess the statistical significance between the conditions, Mann‐Whitney U‐tests were applied using Origin® software. Matlab^TM^ was used to analyze the FRAP data and the script is available upon request. A diffusion uncoupled, two‐component model was used to interpret our FRAP results as previously described in (Govindaraj & Post, 2021; Govindaraj et al., 2019; Govindaraj et al., 2024). The method of FRAP acquisitions and its calculations are explained in the supplementary figures S1 and S2, respectively.

### 2.6 Cluster analysis

Hierarchical clustering was used to identify a number of clusters present in the data set. Euclidean clustering algorithm was used. The optimal number of clustering was identified using Dynamic Tree Cut. Cluster analysis was performed using R version 4.2.2 (Langfelder et al., 2008). Among FRAP variables, the immobile fraction was used for cluster segregation. The cluster with a higher immobile fraction, per donor, per condition was assigned Cluster 1 and the cluster with the lower immobile fraction was assigned Cluster 2. Cells of same cluster per treatment, across all donors within the health state, were combined. For example, in the control condition, cluster 1 all three OA donors were combined. Heat‐maps were generated using Origin® software.

### 2.7 Cell cycle synchronization

hPCs (40,000 cells/well) were seeded on glass coverslips in a 24‐well plate in chondrocyte proliferation media. After 1 day cells were washed, and the medium was replaced with chondrocyte proliferation media without FBS. After 24 h, cells were washed and the medium was replaced with chondrocyte proliferation media with FBS. Post synchronization, cells were analyzed at 3 time points, at 0 h (after 24 h FBS free media), 2 h (after 24 h FBS free media followed by 2 h with FBS media), 6 h (after 24 h FBS free media followed by 6 h with FBS media). Cells were transfected with SOX9‐mGFP and were maintained in chondrocyte proliferation media with FBS for 6 h to allow cells to synthesize SOX9‐mGFP and then the usual synchronization cycle was started as described above.

### 2.8 Immunofluorescence staining

hPCs (40,000 cells/well) grown on coverslips within 24‐well plates were fixed with freshly prepared 4% PFA and washed 3x with ice‐cold PBS at 5 min intervals and simultaneously permeabilized and blocked with 0.3% Triton‐X‐100% and 1% BSA in PBS for 15 min. Rabbit anti‐SOX9 antibody (AB5535, Millipore) at 1:50 dilution was used detect SOX9 protein. Cells were incubated with the primary antibody in the blocking and permeabilization solution at 4⁰C for overnight. Coverslips with cells were washed 3x with PBS. Goat anti‐rabbit AF568 antibody (ab175471, Abcam) at the dilution of 1:500 was used to detect the primary anti‐SOX9 antibody. Coverslips with cells were washed 3x with PBS and mounted on a microscopic glass side with VectaShield containing DAPI. Cells stained for SOX9 were imaged using a Nikon A1 confocal microscope with a 60x/1.2 NA water immersion objective. The axial resolution of the confocal images is 900 nm.

### 2.9 mRNA isolation and RT‐qPCR

hPCs (80,000 cells/well) were plated in a 12‐well plate, and cytokines and inhibitors treatments were given for 24 h at the concentrations mentioned above. For treatments with inhibitors and cytokines, cytokines were added after 1 h pretreatment with the inhibitors, and the 24 h time point was calculated from the time cytokines were added. mRNA was isolated using either NucleoSpin RNA isolation kit (740955, Macherey‐Nagel) or TRIzol^TM^ (15596026, Invitrogen), and linear acrylamide as a co‐precipitant and the manufacturer protocol was followed. Purity and concentration of RNA samples were measured by Nanodrop 2000. cDNA was synthesized from total RNA with iScript cDNA synthesis kit (Bio‐Rad). Real‐time PCR analysis was carried out using SYBR Green mix (Bioline) in a Bio‐Rad CFX‐100 RT‐PCR. Gene expression is reported as the relative mRNA expression (Livak & Schmittgen, 2001) and is normalized to untreated control and GAPDH. Primer sequences are specified in the supplementary information (Table S2).

## 3 RESULTS

### 3.1 Explanation of FRAP parameters

A brief illustration of FRAP method is presented in Figure S1 and we refer the readers to (Govindaraj & Post, 2021; Govindaraj et al., 2019) for more details. A schematic FRAP curve with calculations is presented in Figure S2. FRAP terminologies used: Immobile Fraction (IF) is the fraction of SOX9‐mGFP bound to DNA, and this parameter is used to denote SOX9 activity. A_1_ is the unbound fraction of SOX9‐mGFP that recovers immediately after photobleaching. A_2_ is the fraction of SOX9‐mGFP that is bound to DNA. Recovery half‐time (t½) of A_1_ and A_2_ denote half of the time required for fluorescence recovery of A_1_ and A_2_ populations, respectively. A_1_/A_2_ is the ratio of molecules in the A_1_ to A_2_ populations.

### 3.2 SOX9‐DNA binding is lower in preserved and OA hPCs as compared to healthy hPCs

**Figure 1:**
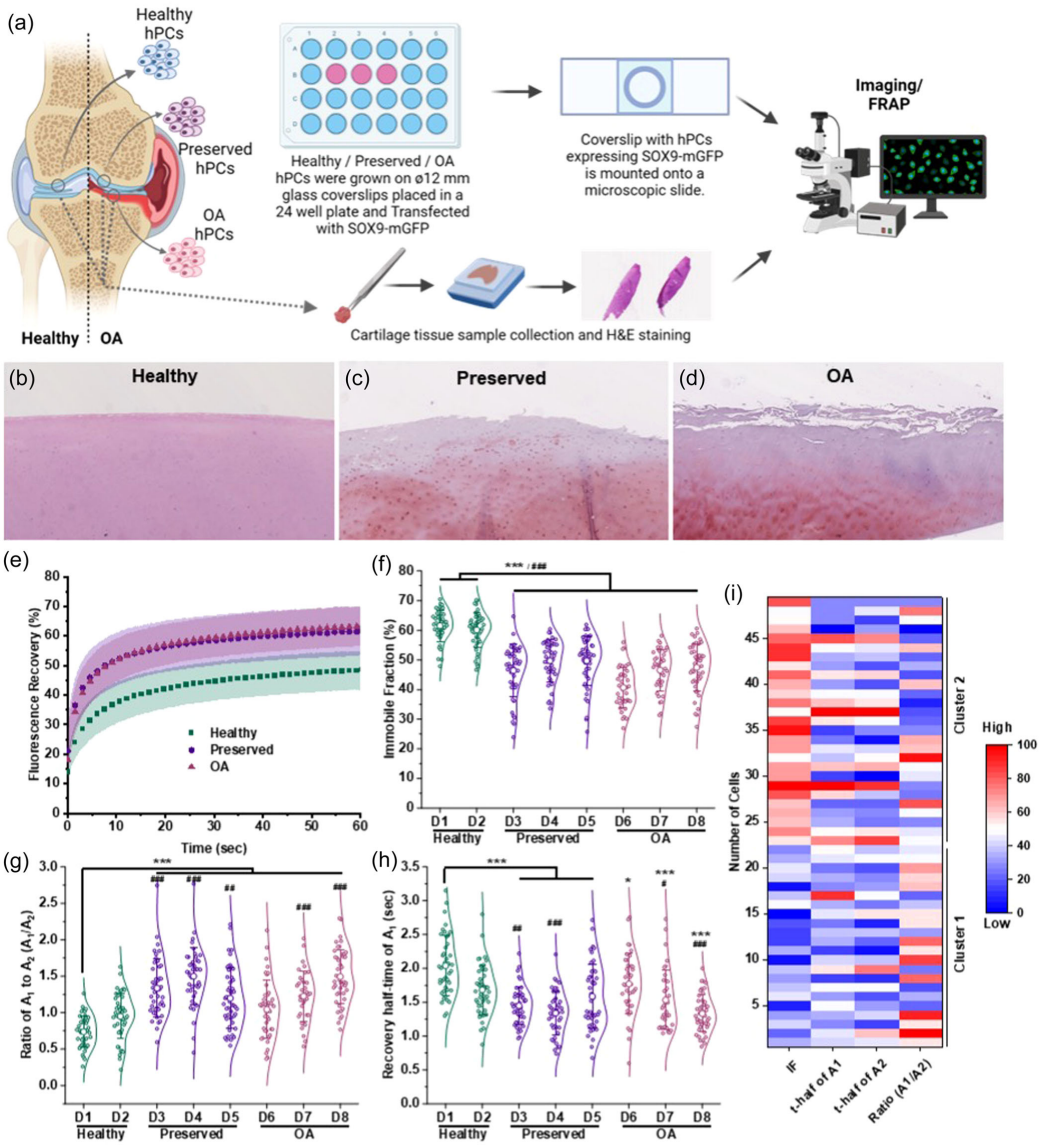
Steady state FRAP measurements show that SOX9‐mGFP binding to DNA is lower in preserved and OA hPCs as compared to healthy hPCs. (a) Schematic diagram showing hPCs isolation and experimental methods to characterize the tissue and quantify SOX9 mobility. H&E staining on the cartilage tissue sections shows the morphology of (b) healthy (c) preserved (d) OA tissues. (e) Averaged FRAP curves show SOX9‐mGFP mobility in healthy (*n* ≥ 76), preserved (*n* ≥ 120), and OA hPCs (*n* ≥ 120). Shades indicate standard deviation (SD). (f) Immobile fraction, (g) Ratio of fast‐diffusing population (A_1_), (h) Recovery half‐time of A_2_ of SOX9‐mGFP in the healthy (*n* ≥ 42, per donor), preserved (*n* ≥ 38, per donor) and OA hPCs (*n* ≥ 36, per donor). D = Donor. Mann‐Whitney U‐test was used for statistical analysis. Statistical significance was calculated between the healthy donors and the preserved and OA donors as stated. Whiskers indicate SD. (i) Heat‐map shows cluster analysis of a healthy donor using FRAP variables. Mann‐Whitney U‐test, */^#^ *p* < 0.05, **/^##^ *p* < 0.01, ***/^###^ *p* < 0.001. * and ^#^ = compared to healthy donor 1 and 2 respectively.

SOX9 binding at the regulatory elements, such as promotor and enhancer regions of its target genes, is essential for their expression (Govindaraj et al., 2019). Based on the analysis of SOX9 target genes, it is known that SOX9 transcriptional activity is decreased in preserved and OA hPCs as compared to healthy hPCs (Zhang et al., 2015). Since impaired SOX9 function is implicated in OA pathology, to understand the changes in SOX9 activity during OA progression, we isolated hPCs from healthy, preserved, and OA cartilage (Figure 1a‐d) and quantified SOX9‐DNA binding. FRAP measurements of steady‐state SOX9 dynamics revealed that the mobility of SOX9 is significantly higher in preserved and OA hPCs as compared to healthy hPCs (Figure 1e). We did not observe a difference in mobility between preserved and OA hPCs. Consequently, that resulted in a significantly lower SOX9 immobile fraction (fraction bound to DNA) in preserved and OA hPCs (Figure 1f and Table 1). Moreover, the ratio of fast‐moving (A_1_; unbound) fraction of SOX9 was higher and their recovery half‐times were shorter in those hPCs (Figure 1g,h). A quick and 100% recovery of mGFP protein (without SOX9) indicates that the mGFP‐tag on SOX9 does not play any role in the binding of SOX9‐mGFP on DNA (Figure S3). These data indicate that the SOX9‐DNA binding is lower and is highly dynamic in the hPCs present in the diseased joint. Interestingly, the violin plots indicated the presence of clusters in the FRAP dynamics data. Subsequent cluster analysis of SOX9 dynamics data of a donor indicated the presence of at least two clusters (Figure 1i) and we further performed cluster analysis in the FRAP data to understand SOX9 dynamics in relation to OA pathology.

**Table 1:** Averaged FRAP rates of SOX9‐mGFP, per healthy, preserved and OA donors.

hPCs	Immobile Fraction (%)	Ratio (A_1_/A_2_)	Recovery Half‐time of A_1_ (sec)	Recovery Half‐time of A_2_ (sec)
Healthy	60.82 ± 5.53	0.85 ± 0.29	1.85 ± 0.44	15.04 ± 3.92
Preserved	49.14 ± 8.02	1.31 ± 0.38	1.48 ± 0.38	13.34 ± 3.56
OA	46.29 ± 8.55	1.17 ± 0.36	1.65 ± 0.47	14.24 ± 4.1

### 3.3 Anabolic factors and catabolic inhibitors increase SOX9‐DNA binding in preserved and OA hPCs

**Figure 2:**
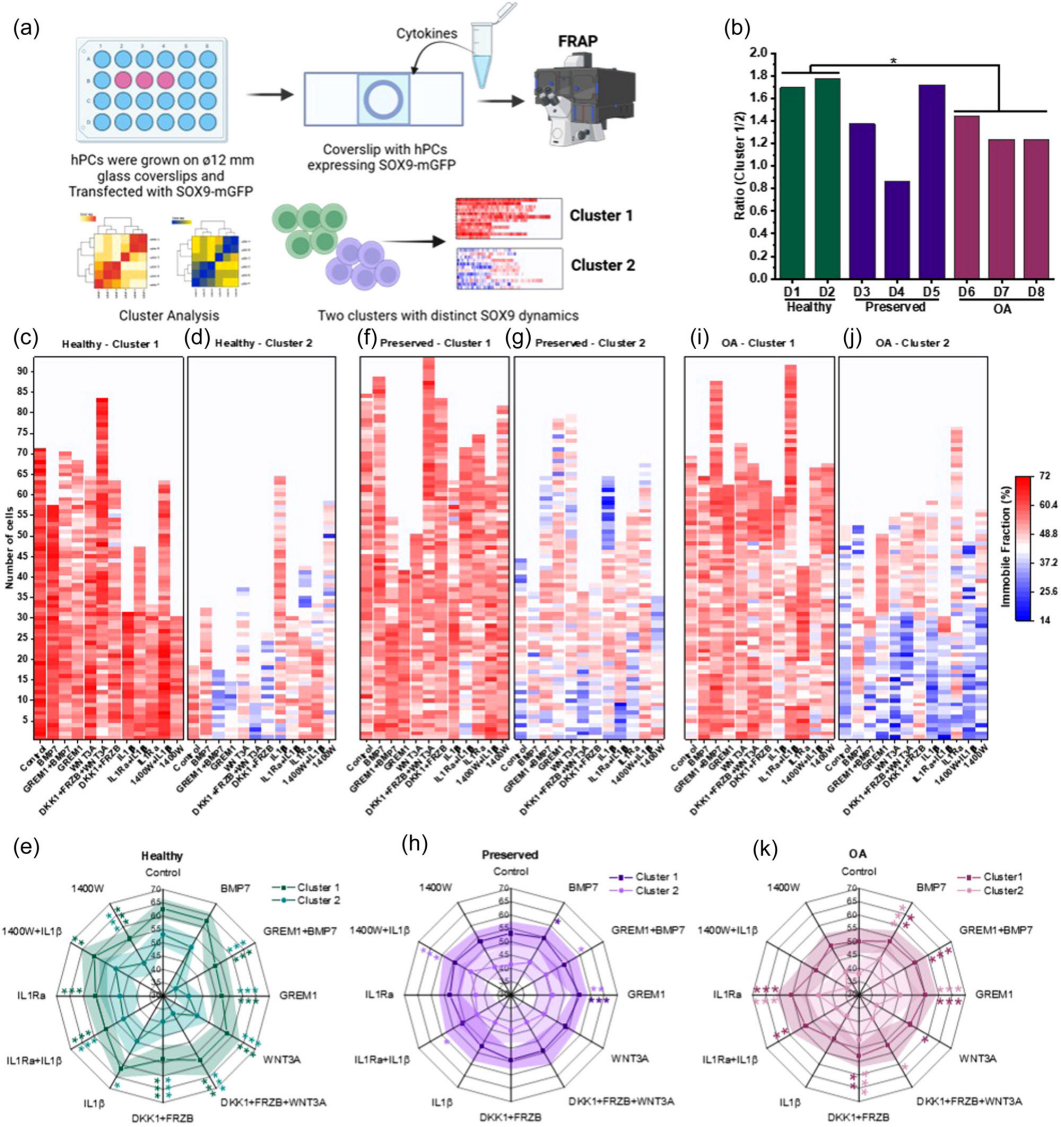
hPCs contain two clusters of cells with distinct SOX9 dynamics in response to extracellular stimuli. (a) Schematic diagram shows experimental methods, and the cluster analysis shows presence of two clusters of cells in hPCs. (b) Ratio of cells in cluster 1 to cluster 2. (c‐k) Heat‐maps at the single cell level and spider plots at the population level show SOX9 immobile fraction in cluster 1 and 2 of healthy (c–e), preserved (f–h) and OA hPCs (i–k), respectively (n = 2 healthy, 3 preserved and 3 OA donors). Statistical significance was calculated between the control and treatments. Mann‐Whitney U‐test, *p < 0.05, **p < 0.01, ***p < 0.001.

Extracellular signaling factors are known to influence SOX9 activity (Sahu et al., 2020). To map the factors that regulate SOX9 transcriptional dynamics, we stimulated hPCs with BMP (anabolic), WNT (catabolic), and IL1β (inflammatory) pathway activators and their inhibitors. FRAP quantification of SOX9 dynamics and subsequent cluster analysis revealed that the hPCs had two distinct clusters of SOX9 dynamics (Figure 2a). The cluster with a higher SOX9‐DNA binding was assigned cluster 1 and the cluster with a lower SOX9‐DNA binding was named cluster 2. Interestingly, the ratio of cells with higher SOX9‐DNA binding was consistently lower in preserved and OA donors as compared to healthy donors (Figure 2b).

As expected, the heat‐map at the single cell level resolution shows that SOX9‐DNA binding was highest in cluster 1 of healthy hPCs (Figure 2c). Moreover, both clusters of healthy hPCs were very sensitive to cytokine stimulations (Figure 2c,d,e, and Table S3). BMP7 inhibitor GREM1, WNT3A and its inhibitors DKK1 and FRZB, IL1β and its inhibitor IL1Ra, and iNOS inhibitor 1400W significantly decreased SOX9‐DNA binding in both or either of the clusters of healthy hPCs (Figure 2e). In contrast, the anabolic factor BMP7 neither decreased nor increased SOX9‐DNA binding in both clusters of healthy hPCs. The ratio of unbound SOX9 was also lowest in the healthy hPCs, and external stimuli significantly increased the ratio of unbound SOX9 in both clusters (Figure S4 A–C and Table S4). Recovery half‐times of unbound (A_1_) and bound (A_2_) SOX9 were also longest in both clusters of the healthy hPCs, and they significantly decreased upon addition of external stimuli (Figure S5 A–C, S6 A‐C, and Table S5 and S6). These data indicate that not only SOX9‐DNA binding was highest, SOX9 residence time on DNA was also longest in the healthy hPCs. Extracellular signaling imbalance significantly decreased SOX9‐DNA binding and its residence time on DNA.

Preserved hPCs were comparatively less responsive to extracellular stimuli and SOX9‐DNA binding was significantly lower than healthy but higher than OA hPCs (Figure 2f‐h and Table S3). Only BMP7 and GREM treatments significantly increased SOX9‐DNA binding in the cells of cluster 1. GREM1 + BMP7, GREM1, IL1Ra + IL1β, and 1400W + IL1β treatments increased SOX9‐DNA binding in cells of cluster 2 (Figure 2h), which was statistically significant, as indicated. This suggests that in the presence of inhibitors, stimulants do not affect intracellular signaling to decrease SOX9 DNA binding. The ratio of unbound SOX9 was the highest in the preserved hPCs (Figure S4 D‐F and Table S4). Only treatment with the iNOS inhibitor 1400W significantly increased the ratio of unbound SOX9 in cluster 1 of preserved hPCs (Fig. S4F). BMP7, WNT3A, and IL1Ra treatments significantly decreased the ratio of unbound SOX9 in cluster 2 of preserved hPCs. Interestingly, BMP7, GREM1, and the inhibitors of catabolic and inflammatory signals increased SOX9 DNA residence times in either or both clusters (Figure S5D‐F, S6D‐F, Table S5 and S6). These data suggest that hPCs in a joint with trauma may be less sensitive to external stimuli and anabolic factors and that use of inhibitors or antagonists of catabolic and inflammatory mediators can increase SOX9 signaling and thus cartilage homeostasis.

OA hPCs were more responsive to external stimuli as compared to preserved hPCs (Figure 2i‐k and Table S3). Since SOX9 activity was already the lowest in the OA hPCs, treatment with catabolic or inflammatory mediators did not further decrease SOX9‐DNA binding (Figure 2i‐k). In contrast, the anabolic factor BMP7 and its inhibitor GREM1, as well as inhibitors of catabolic and inflammatory pathways DKK1 + FRZB and IL1Ra, respectively, increased SOX9‐DNA binding in either or both clusters (Figure 2k). Only BMP7, GREM1, DKK1 + FRZB, and IL1Ra treatments significantly decreased the ratio of unbound over bound SOX9 in both clusters (Figure S4 G‐I and Table S4). Interestingly, with exception of GREM1 and IL1β treatments, BMP7 and the inhibitors of catabolic, inflammatory, and iNOS pathways decreased the recovery half‐time of unbound SOX9 in one or both clusters of OA hPCs (Figure S5 G‐I and Table S5). Treatments with the anabolic factor BMP7 along with its inhibitor GREM1, catabolic factor WNT inhibitor DKK1 + FRZB, inflammatory factor IL1β inhibitor IL1Ra, and iNOS inhibitor 1400W significantly increased SOX9 DNA residence times. Together, these data suggest that anabolic factors and inhibitors of catabolic mediators might facilitate SOX9 binding and its residence time on DNA in OA hPCs. Moreover, based on SOX9‐DNA binding and its residence time on DNA, it appears that SOX9 activity may be higher in the hPC subpopulation of cluster 1 as compared to the subpopulation of cluster 2.

### 3.4 Nuclear localization patterns of SOX9 correlate to its dynamics

**Figure 3:**
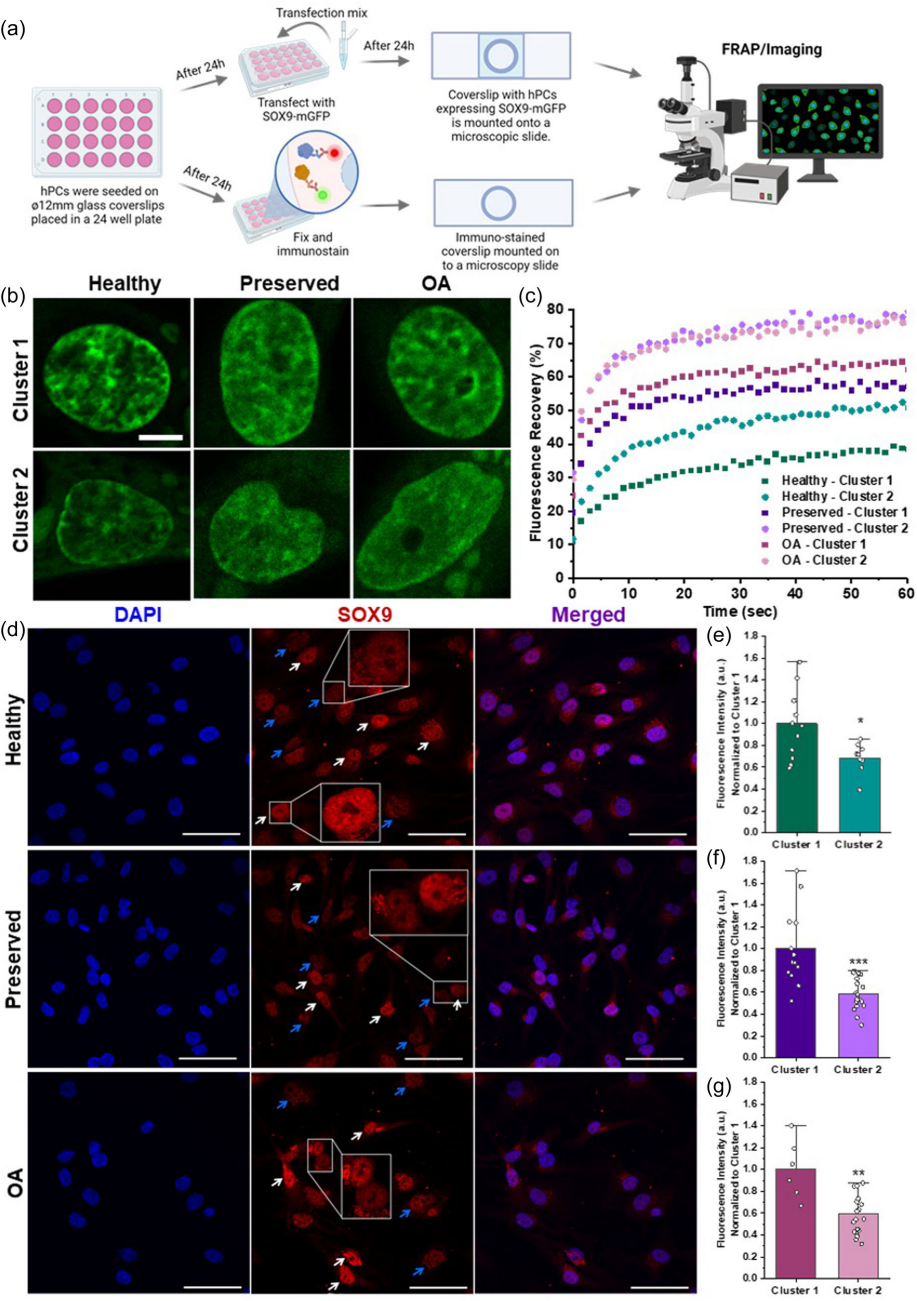
Nuclear localization patterns of SOX9(‐mGFP) correlate to its dynamics. (a) Schematic diagram showing experimental methods. (b) Healthy, preserved, and OA hPCs expressing SOX9‐mGFP show that cluster 1 and 2 have distinct nuclear SOX9 localization patterns and (c) corresponding FRAP dynamics. Scale bar: 5 μm. (d) Immunostaining of endogenous SOX9 shows presence of two distinct SOX9 expression levels in healthy, preserved, and OA hPCs and corresponding fluorescence intensity quantifications (e–g), white arrows indicate high intensity discreet nuclear localization (cluster 1) and blue arrows indicate low intensity diffused nuclear localization (cluster 2). Scale bar: 100 μm. Mann‐Whitney U‐test, *p < 0.05, **p < 0.01, ***p < 0.001.

We asked whether there is a correlation between the nuclear localization pattern of SOX9 and its dynamics between the two clusters or among the health state of hPCs. We quantified SOX9 dynamics using FRAP in steady‐state (without the addition of external stimuli) as well as using immuno‐stained endogenous SOX9 to answer this question (Figure 3a). Surprisingly, the majority of cells in cluster 1 showed a discrete nuclear localization pattern in healthy hPCs and to a lesser extent in preserved and OA hPCs. In contrast, the majority of cells in cluster 2 showed a diffused nuclear localization pattern in healthy, preserved, and OA hPCs (Figure 3b and S7‐S9). SOX9 dynamics data as measured by FRAP corroborated with the nuclear localization patterns of SOX9. Regardless of the health state, cells in cluster 1 with a discrete nuclear localization pattern showed lower SOX9 dynamics as compared to cluster 2 (Figure 3c). This data indicates that cells with discrete SOX9 foci in the nucleus will have a higher SOX9 binding and a longer DNA residence time as compared to cells with a diffused nuclear localization pattern.

To rule out the overexpression of SOX9 playing any role in the presence or absence of a distinct nuclear localization pattern and dynamics, we immuno‐stained endogenous SOX9 in untransfected hPCs (Figure 3d). Immunofluorescence confirmed that, regardless of the health state, there are at least two types of hPCs present in the cartilage tissue, one expressing higher levels of SOX9 (cluster 1) and other with lower levels of SOX9 (cluster 2). Moreover, fluorescence intensity calculations confirmed that the SOX9 expression levels are significantly different between cells with discrete and diffused SOX9 nuclear localization in healthy (Figure 3e), preserved (Figure 3f), and OA (Figure 3g) hPCs. Immunostaining of endogenous SOX9 in cell‐cycle synchronized hPCs confirmed that these distinct nuclear localization patterns were not a result of the cell cycle (Figure S10).

### 3.5 SOX9 target gene expression correlates to SOX9 dynamics

**Figure 4:**
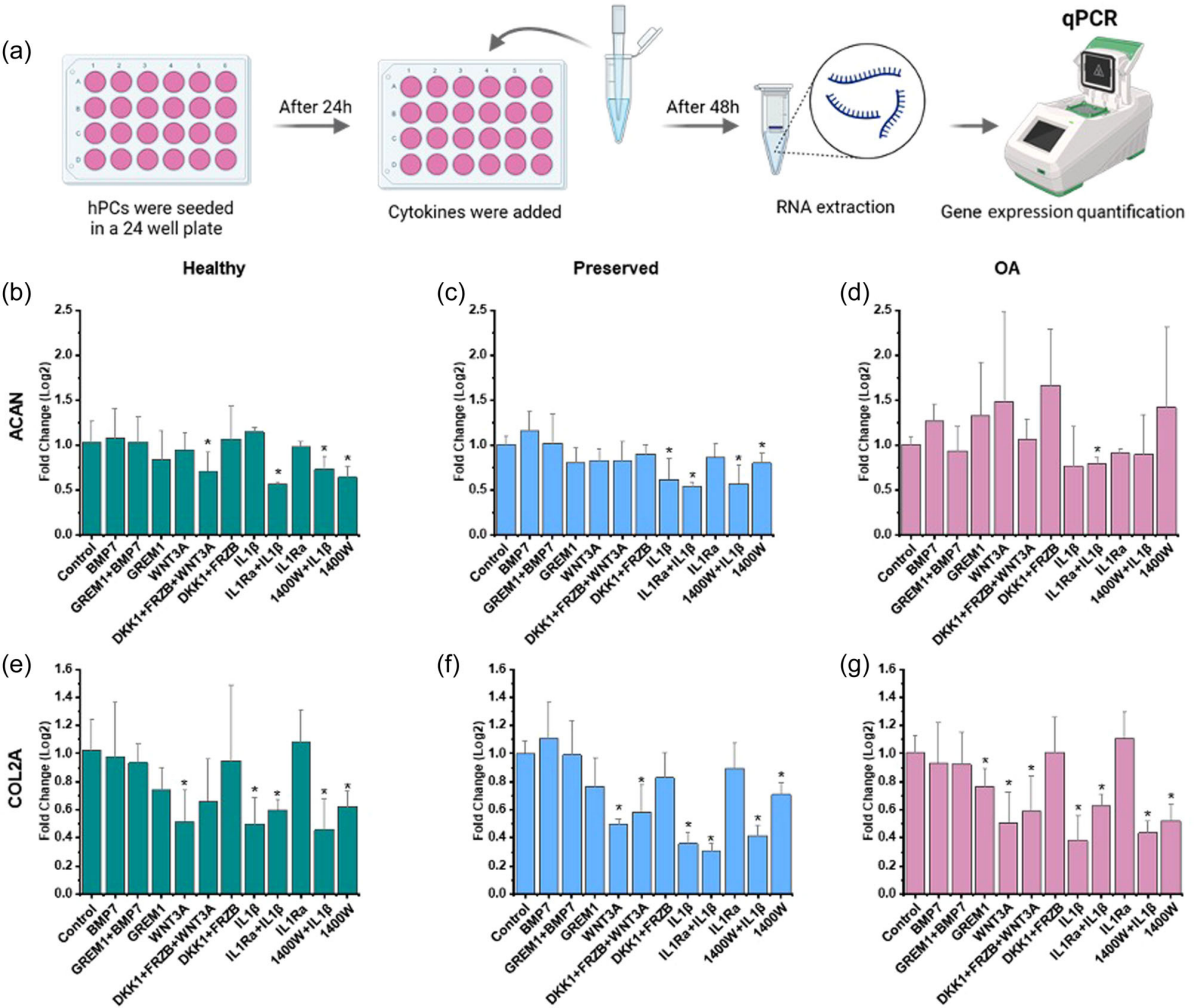
ACAN and COL2A gene expression correlate to SOX9‐DNA binding in healthy, preserved and OA hPCs. (a) Schematic diagram shows experimental methods. (b–d) ACAN and (e–g) COL2A expression in healthy, preserved and OA hPCs in response to cytokine treatments. mRNA expression is normalized to GAPDH and untreated control. *n* = 2 healthy, 3 preserved and 3 OA donors. Student t‐test, significance is calculated between control and the indicated treatment, *p < 0.05.

SOX9‐DNA binding is known to regulate its target genes expression (Govindaraj et al., 2019). To shed more light on the correlation between SOX9 dynamics and its transcriptional activity, we quantified the expression of SOX9 target genes – *COL2A* and *ACAN* – in response to external stimuli, (Figure 4a‐g). Mostly, changes to the gene expression pattern in response to external stimuli was similar among healthy, preserved, and OA hPCs. Regardless of the health state of hPCs, BMP7 increased *ACAN* and *COL2A* expression, however, the increase was not significant (Figure 4b‐d). In healthy hPCs, treatment with WNT3A and IL1β and/or their inhibitors decreased expression of either *ACAN* or *COL2A* or both (Figure 4b,e). Notably, in preserved and OA hPCs, WNT inhibitors DKK1 + FRZB and IL1 inhibitor IL1Ra treatments did not change *ACAN* and *COL2A* expression (Figure 4c,d,f,g). Inhibitor treatments increased *ACAN* expression in OA hPCs, however, the increase was not significant (Figure 4d). Expression of *COL2A* was lower in preserved and OA hPCs (high Ct values) as compared to that in healthy hPCs (Figure S11). Together, these data indicate that these stimulations have influenced SOX9 target gene expression in hPCs.

## 4 DISCUSSION

Cellular signaling plays a central role in maintaining cell and tissue homeostasis and understanding signaling interplay sheds light on possible therapeutic targets. SOX9 is the master regulator of chondrocyte and cartilage homeostasis, and its impaired function is implicated in cartilage disease. Studies investigating transcription factor activity are often performed at the population level, lacking spatial resolution. Our SOX9 dynamics data at the single cell level has uncovered its differential dynamics among subpopulations within the cell type.

SOX9 activity is known to be downregulated during OA pathophysiology. This is evident from the decreased expression of *SOX9* mRNA and its target genes and proteins, such as *COL2A* and *ACAN* in OA hPCs (Lee & Im, 2011). Epigenetic modifications in OA hPCs, which limits the interaction of transcription machinery, also reportedly play a key role in the downregulation of SOX9 and its target gene and protein expression (Kim et al., 2013; Ramos & Meulenbelt, 2017). Our quantitative FRAP measurements indicate that the amount of SOX9‐DNA binding and its residence time on DNA is considerably reduced in preserved and OA as compared to healthy hPCs. This results in the increase of ratio of unbound over bound SOX9‐mGFP in chondrocytes from deceased joint. Altered epigenetic modifications in OA hPCs – which either mask the binding sites of SOX9 in the DNA or change SOX9 molecular state – likely to be one of the causes of reduced SOX9 transcriptional activity. There are several reports indicating differences between gene expression profile of hPCs isolated from minimally damaged area (preserved) and highly damaged (OA) area of OA knee (Dell'Accio et al., 2008; Snelling et al., 2014). In line with those reports, our data indicate that the SOX9‐DNA binding is significantly lower in the hPCs isolated from preserved, and OA cartilage tissues.

Primary articular chondrocytes are known to be the only cell type present in the cartilage tissue (Benedek, 2006). However, several recent reports claiming chondrogenic heterogeneity with distinct cellular phenotypes (Fan et al., 2024; Hu et al., 2022). Mobility of SOX9‐mGFP, measured in hPCs showed at least two distinct FRAP rates as identified by cluster analysis, in healthy, preserved, and OA hPCs. This potentially indicates two differential SOX9 transcriptional activity between these two sub‐populations of hPCs. Surprisingly, these cells differentially respond to the growth factors in the microenvironment. BMP7, already used in an OA clinical trial (Badlani et al., 2009), seems to enhance SOX9‐DNA binding in OA hPCs. WNT3A and IL1β, implicated in OA progression and known to reduce SOX9 transcriptional activity (Govindaraj et al., 2019), decreased SOX9 binding in healthy hPCs and did not alter it in OA hPCs. This explains that more SOX9 is bound to DNA in healthy hPCs and that it is affected by these catabolic factors, whereas OA hPCs were already exposed to these factors during OA progression and SOX9‐DNA binding and activity is lower in these cells.

Spatiotemporal resolution of transcription factor dynamics captures the relationship between spatial arrangement of SOX9‐mGFP inside the nucleus and its mobility. Nucleus with discrete and sharp SOX9‐mGFP foci leads to higher transcriptional activity implies strong DNA binding exerts higher expression of target genes. Overexpression of SOX9 in OA chondrocytes has been shown to be a promising approach to revert to healthy phenotype (Rey‐Rico et al., 2018; Tao et al., 2017; Tew et al., 2005). However, it is unclear how altered epigenetic changes in OA play a role in these studies. Our SOX9‐mGFP overexpression data show a clear difference in nuclear localization pattern between healthy and OA hPCs and its relationship in DNA binding. We show here that SOX9 overexpression in preserved and OA hPCs did not increase SOX9‐DNA binding to the levels found in healthy hPCs. It is of note that we currently do not know how these cluster 1 and 2 cells are distributed in the cartilage. Are they randomly distributed, present in certain areas, or are the cells present in distinct cartilage zones. However, it can be reasonably argued that cells present in different zones may respond distinctly to the external stimuli as they are phenotypically different (Coates & Fisher, 2010).

In conclusion, we identified two subpopulations within hPCs. Each subpopulation shows distinct SOX9 dynamics and responds differently to external stimuli. SOX9 binding and SOX9 residence time on DNA are inherently higher in healthy hPCs as compared to preserved and OA hPCs, correlating to a lower sensitivity to external stimuli in preserved and OA hPCs. Furthermore, we have observed a correlation between SOX9 dynamics and its nuclear localization pattern. Our findings provide new insights into transcription factor dynamics and their functional relevance and offer valuable insights into the role of SOX9 in osteoarthritis pathology.

## AUTHOR CONTRIBUTIONS

Kannan Govindaraj: Experimental planning, Data acquisition, Analysis, Interpretation, and wrote the manuscript. Sakshi Kannan: Performed experiments and data analysis. Rodrigo Coutinho de Almeida and Ingrid Meulenbelt: Performed cluster analysis and wrote related methods section. Lucas Jansen Klomp: wrote Matlab scripts to sort data. Janine N. Post: Conceptualization of idea, Interpretation of data, Supervision, co‐wrote and reviewed the manuscript. Marcel Karperien: Supervision, critically reviewed the manuscript.

## CONFLICT OF INTEREST STATEMENT

No, there is no conflict of interest.

## Supporting information

Supporting information.

Supporting information.
