# Supplementary material for: Dissecting SOX9 dynamics reveals its differential regulation in osteoarthritis
Source: J Cell Physiol. 2024 Sep 29;239(12):e31443. doi: 10.1002/jcp.31443 (PMC11649970; doi:10.1002/jcp.31443)
Supplement: Supplementary file 1 — Supporting information. [file JCP-239-0-s002.docx]

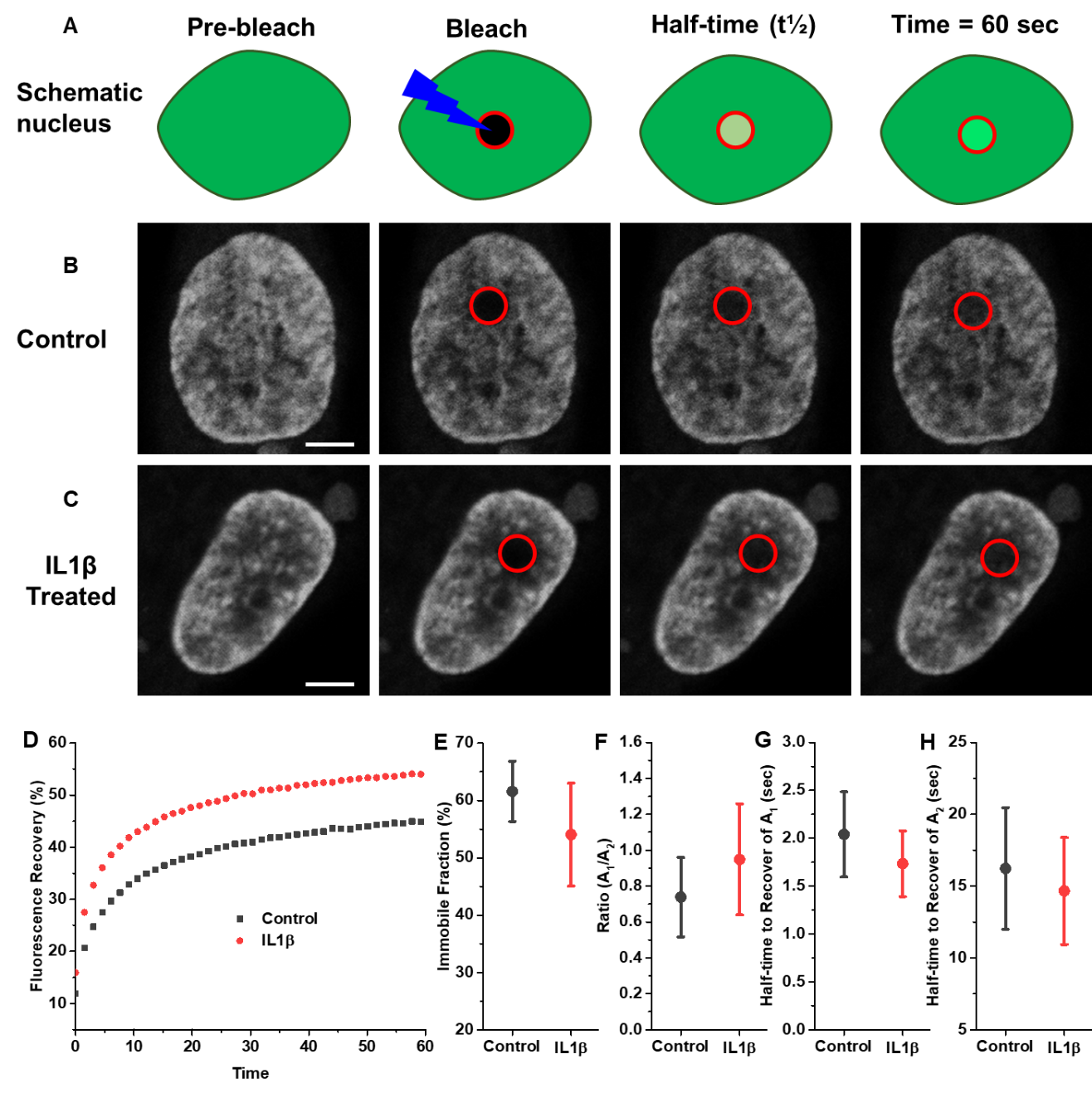


***Figure S1. Explanation of the FRAP method and parameters.*** *(A) Schematic diagram illustrating the FRAP method. A small circular region (2.9 μm, Ø) of a nucleus expressing a fluorescent protein is photo-bleached using a high-intensity laser and the recovery of the fluorescence is recorded by time-lapse imaging over a specific period, in our case 4 frames per second, for 60 seconds. (B) Fluorescence recovery of SOX9-mGFP without any treatment. (C) Fluorescence recovery of SOX9-mGFP after IL1β treatment. (D) FRAP curves show the mobility of SOX9-mGFP was increased in the presence of IL1β as compared to the control. This resulted in a lower immobile fraction (E), increased ratio of fast-diffusing population (F), decreased recovery half-time of A_1_ (G) and A_2_ (H). Bar size: 5 μm.*


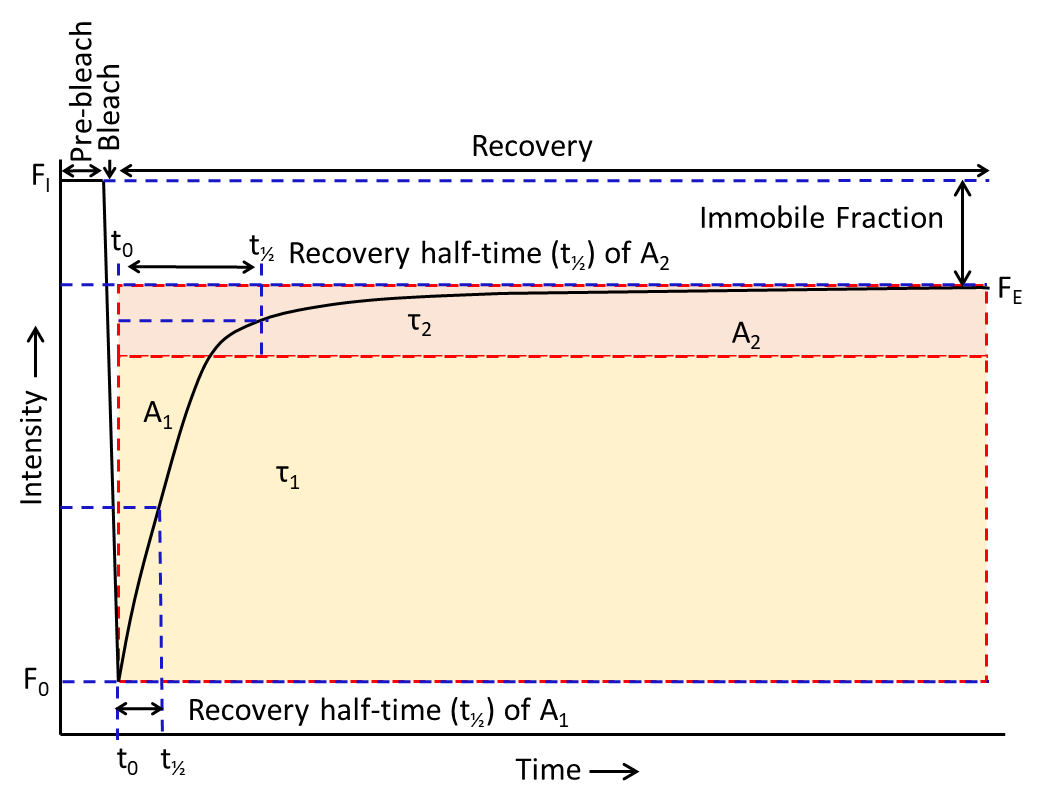


**Figure S2. Schematic representation of a FRAP curve and explanation of parameters.** More than one reaction can contribute to the FRAP recovery. For example, if two reactions, – namely, fast and slow reactions – contribute to FRAP recovery and if these two processes occur in two different time scales, the FRAP curve can be split into two phases (A_1_ and A_2_) as shown. F_I_: Initial intensity, F_0_: Intensity at time point t_0_ (first post-bleach intensity), F_E_: End value of the recovered intensity, t½: Recovery half-time, Immobile fraction is the population of SOX9-mGFP bound to DNA. A_1_ is the amplitude of fast diffusing population of SOX9-mGFP, which is weakly interacting to DNA and contributes to a quick recovery. A_2_ is the amplitude of slow diffusing population of SOX9-mGFP, which strongly interacts on the various binding sites in the DNA.

**Fig. S3. FRAP of mGFP in hPCs (n = 18 cells).**


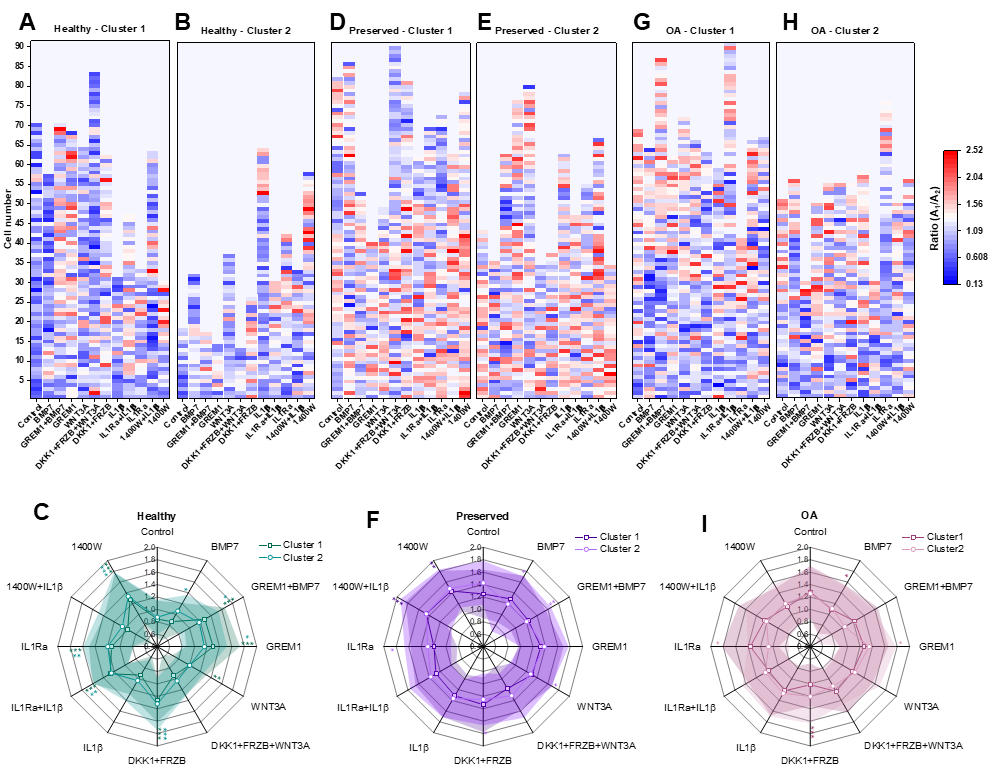


**Fig. S4. Ratio of unbound SOX9 was lower in healthy hPCs.** Heat-maps show the ratio of unbound SOX9 in (A) cluster 1 and (B) cluster 2 of healthy hPCs at the single cell level. (C) Spider plot shows averaged ratio of unbound SOX9 per treatment for both the clusters of healthy donors (n = 2 donors) at the population level. Heat-maps show the ratio of unbound SOX9 in (D) cluster 1 and (E) cluster 2 of preserved hPCs. (F) Spider plot shows averaged ratio of unbound SOX9 per treatment for both the clusters of preserved donors (n = 3 donors). Heat-maps show the ratio of unbound SOX9 in (G) cluster 1 and (H) cluster 2 of OA hPCs. (I) Spider plot shows averaged ratio of unbound SOX9 per treatment for both the clusters of OA donors (n= 3 donors).


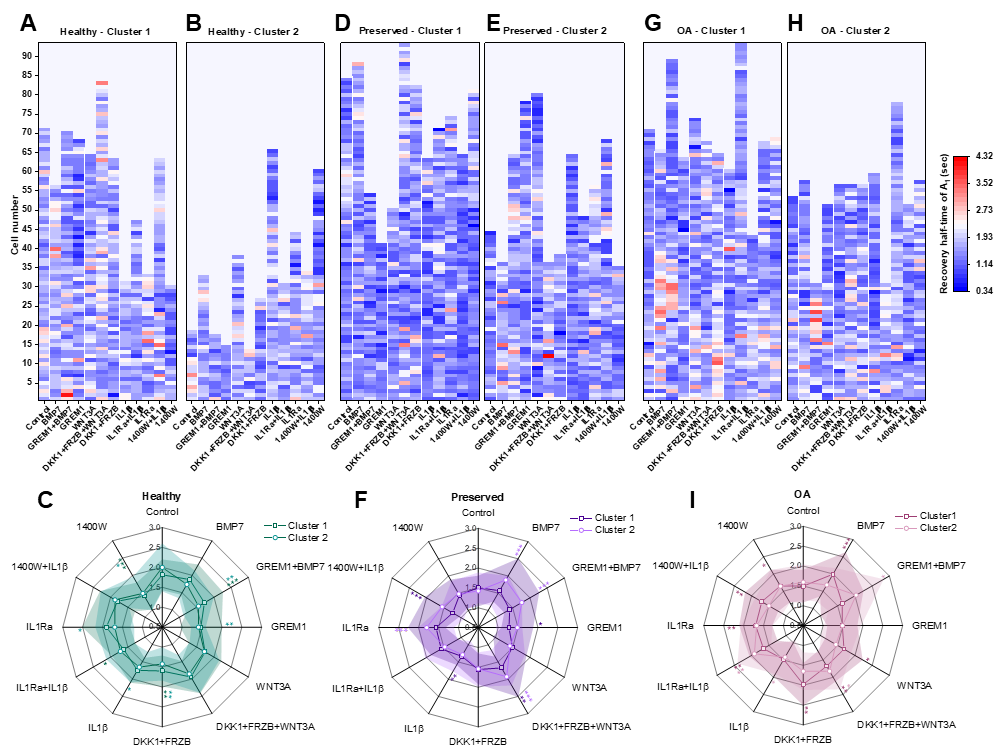


**Fig. S5. Recovery half-time of unbound SOX9 (fraction A_1_) was slowest in healthy hPCs.** Heat-maps at the single cell level show the recovery half-time (sec) of unbound SOX9 in (A) cluster 1 and (B) cluster 2 of healthy hPCs. (C) Spider plot at the population level shows averaged recovery half-time of unbound SOX9 per treatment for both the clusters of healthy donors (n = 2 donors). Heat-maps showing the recovery half-time of unbound SOX9 in (D) cluster 1 and (E) cluster 2 of preserved hPCs. (F) Spider plot shows averaged ratio of unbound SOX9 per treatment for both the clusters of preserved donors (n = 3 donors). Heat-maps show the recovery half-time of unbound SOX9 in (G) cluster 1 and (H) cluster 2 of OA hPCs. (I) Spider plot shows averaged recovery half-time of unbound SOX9 per treatment for both the clusters of OA donors (n= 3 donors).


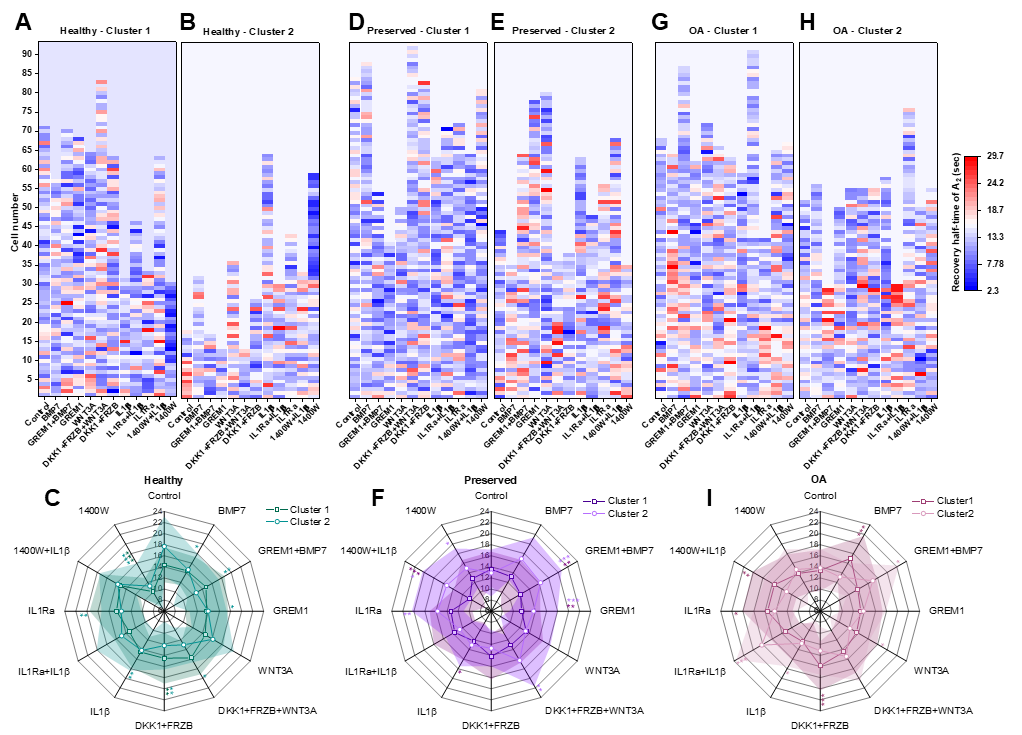


**Fig. S6. SOX9 residence time on DNA (recovery half-time of fraction A_2_) was longest in the healthy hPCs.** Heat-maps at the single cell level show the recovery half-time (sec) of bound SOX9 in (A) cluster 1 and (B) cluster 2 of healthy hPCs. (C) Spider plot at the population level shows averaged recovery half-time of bound SOX9 per treatment for both the clusters of healthy donors (n = 2 donors). Heat-maps show the recovery half-time of bound SOX9 in (D) cluster 1 and (E) cluster 2 of preserved hPCs. (F) Spider plot shows averaged ratio of bound SOX9 per treatment for both the clusters of preserved donors (n = 3 donors). Heat-maps show the recovery half-time of unbound SOX9 in (G) cluster 1 and (H) cluster 2 of OA hPCs. (I) Spider plot shows averaged recovery half-time of bound SOX9 per treatment for both the clusters of OA donors (n= 3 donors).


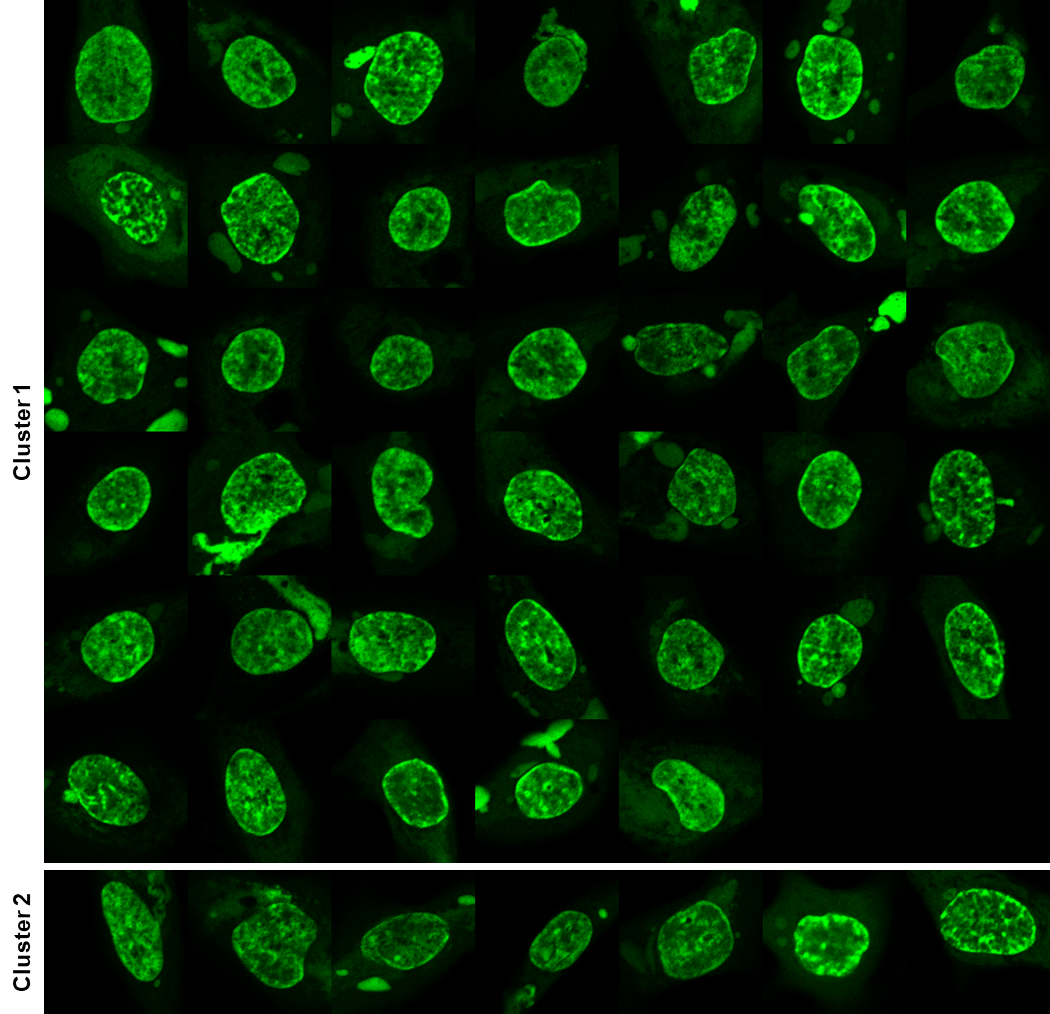


Figure S7. Montage of nuclei showing that the SOX9 nuclear localization pattern is more discreet in cluster 1 and diffuse in cluster 2 of healthy hPCs.


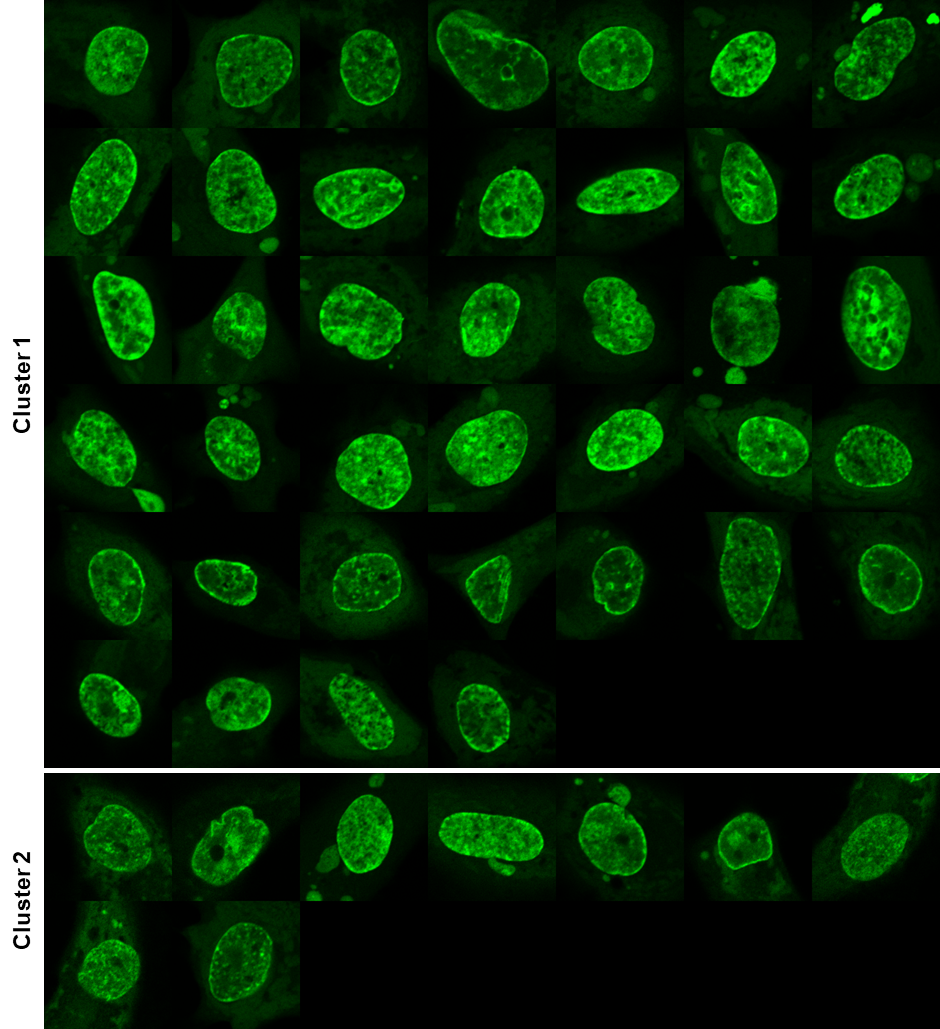


Figure S8. Montage of nuclei showing that the SOX9 nuclear localization pattern is more discreet in cluster 1 and diffuse in cluster 2 of preserved hPCs.


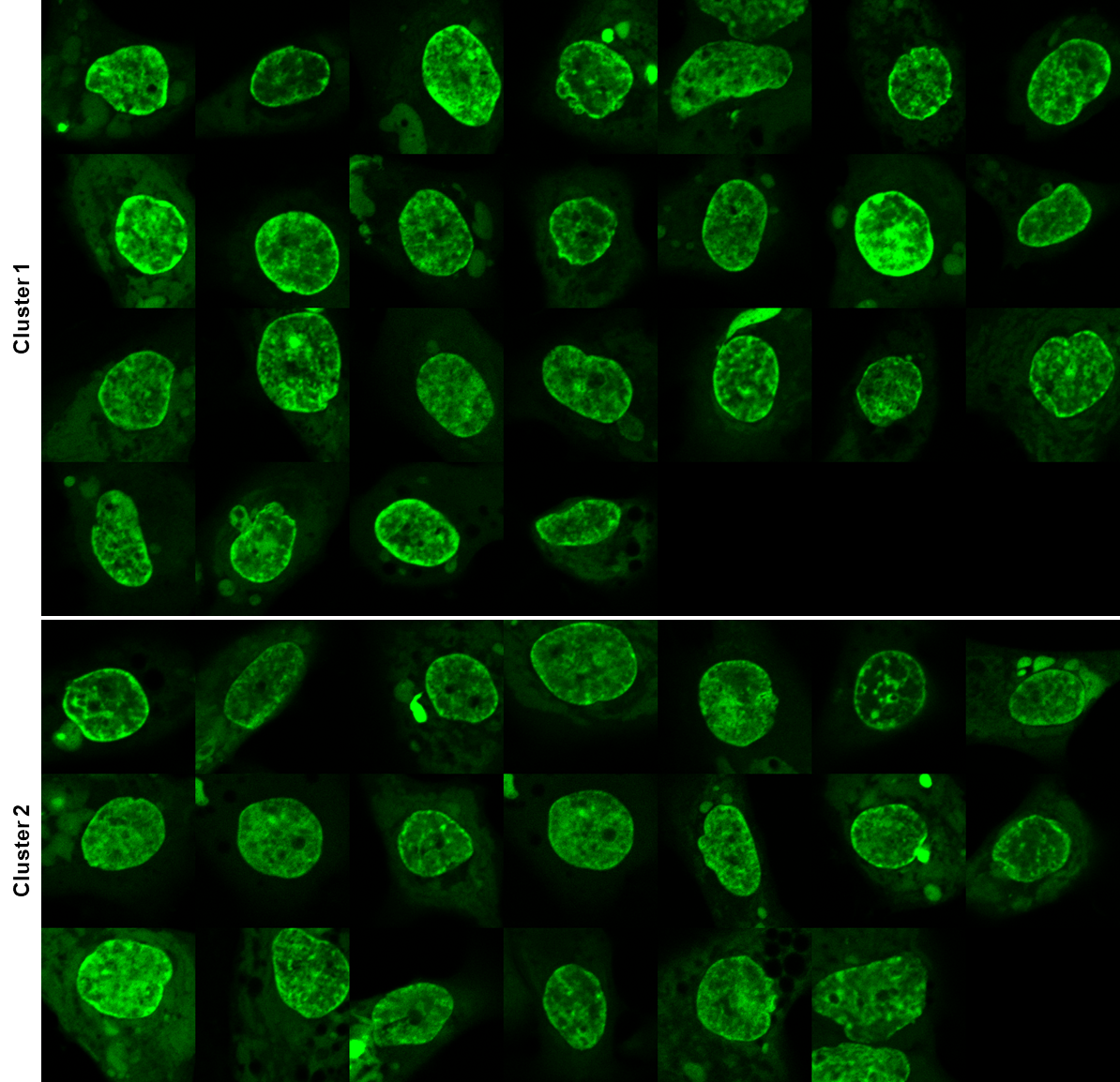


Figure S9. Montage of nuclei showing that the SOX9 nuclear localization pattern is more discreet in cluster 1 and diffuse in cluster 2 of OA hPCs.


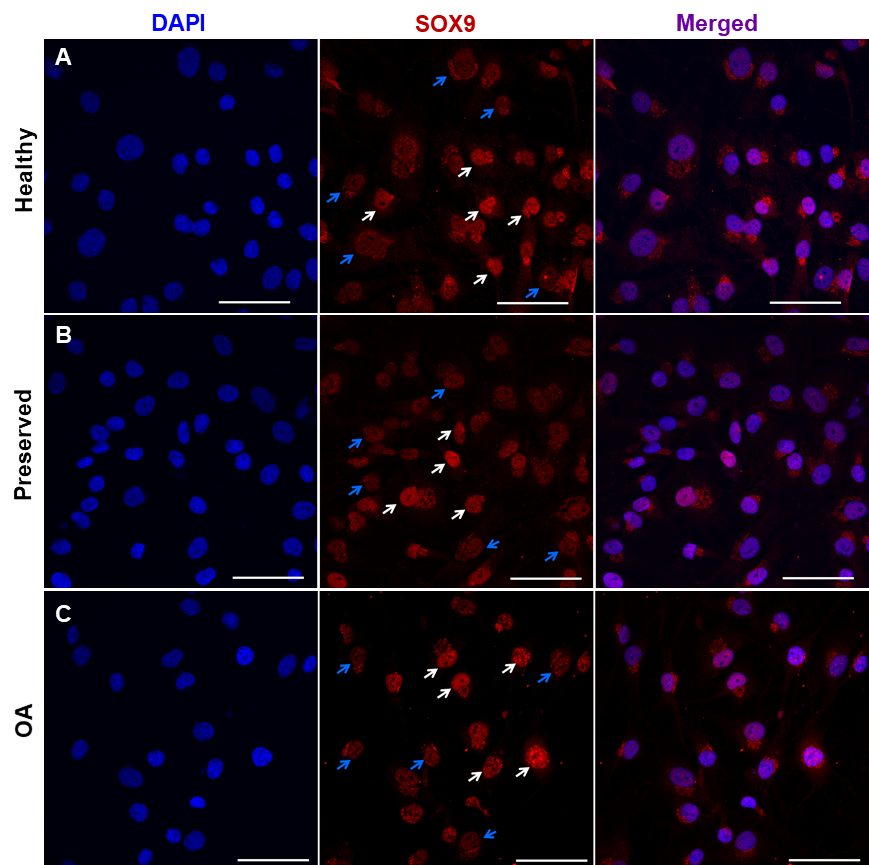


Fig. S10. *Immuno-staining of SOX9 shows presence of two distinct SOX9 expression levels in synchronized (A) healthy, (B) preserved and (C) OA hPCs. hPCs were synchronized for 48 hours.*


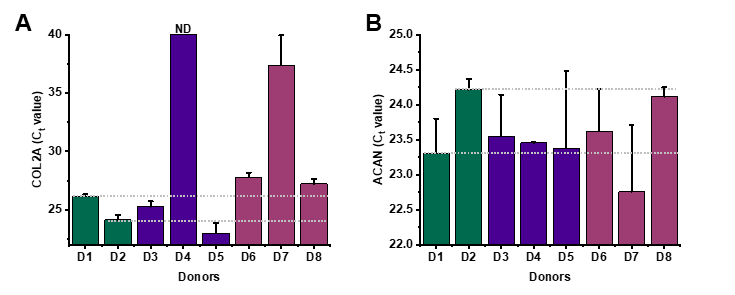


Figure S11. Delta Ct values of *COL2A* and *ACAN* in healthy and OA hPCs in steady state. The higher the Ct value, the lower the gene expression. Lines indicate deviation from healthy donors 1 and 2. A. *COL2A* expression was lower in at least four OA donors as compared to healthy donors. B. *ACAN* expression was lower in five OA donors as compared to healthy donor 1. mRNA concentration: 18.5 ng, ND: Not detected. SD: two technical duplicates were averaged.
