# Supplementary material for: Dissecting SOX9 dynamics reveals its differential regulation in osteoarthritis
Source: J Cell Physiol. 2024 Sep 29;239(12):e31443. doi: 10.1002/jcp.31443 (PMC11649970; doi:10.1002/jcp.31443)
Supplement: Supplementary file 2 — Supporting information. [file JCP-239-0-s001.docx]

***Table S1. Details of donors used in the manuscript.***

| Health State | Donor no. in the manuscript | Age | Gender | OARSI Score |
| --- | --- | --- | --- | --- |
| Healthy | D1 | 26 | M | Not available  (Purchased) |
|  | D2 | 54 | F |  |
| Preserved | D3 | - | - | 2 |
|  | D4 | - | F | 1 |
|  | D5 | - | M | 1 |
| OA | D6 | 66 | F | 3 |
|  | D7 | - | - | 3 |
|  | D8 | 72 | F | Not available |

***Table S2. List of primers used for qPCR.***

| **Target** | **Sequence** |
| --- | --- |
| ACAN | F5' AGGCAGCGTGATCCTTACC 3'  R5' GGCCTCTCCAGTCTCATTCTC 3' |
| COL2A | F5’ CCAGATGACCTTCCTACGCC 3’  R5’ TTCAGGGCAGTGTACGTGAAC 3’ |
| ID1 | F5’ GGCGCTGATCTCGCCGTTGAG 3’  R5’ GCAAGACAGCGAGCGGTGCG 3’ |
| AXIN2 | F5' AGTGTGAGGTCCACGGAAAC 3'  R5' CTGGTGCAAAGACATAGCCA 3' |
| MMP13 | F5' AAGGAGCATGGCGACTTCT 3'  R5' TGGCCCAGGAGGAAAAGC 3' |
| IL1β | F5' TCCCCAGCCCTTTTGTTGA 3'  R5' TTAGAACCAAATGTGGCCGTG 3' |
| GAPDH | F5' CGCTCTCTGCTCCTCCTGTT 3'  R5' CCATGGTGTCTGAGCGATGT 3' |

***Table S3. Percentage of SOX9-mGFP bound to DNA per cluster, per health state of hPCs.***

|  | Healthy | | Preserved | | OA | |
| --- | --- | --- | --- | --- | --- | --- |
|  | Cluster 1 | Cluster 2 | Cluster 1 | Cluster 2 | Cluster 1 | Cluster 2 |
| Control | 62.8 ± 3.8 | 53.1 ± 4.6 | 53.2 ± 4.4 | 41.4 ± 7.8 | 50.3 ± 4.8 | 38 ± 5.2 |
| BMP7 | 62.7 ± 3.4 | 51.1 ± 4.1 | 54.7 ± 4.5 | 44.1 ± 5.9 | 53.2 ± 4.7 | 45.6 ± 7.1 |
| GREM1+BMP7 | 52.8 ± 5.1 | 35.6 ± 4.7 | 53 ± 5.7 | 45.2 ± 6.2 | 54.1 ± 5 | 40.5 ± 7.9 |
| GREM1 | 52.2 ± 4.5 | 39.6 ± 5.3 | 55.9 ± 3 | 45.6 ± 5.9 | 55 ± 4.4 | 45.5 ± 8.1 |
| WNT3A | 57.7 ± 5.6 | 44.8 ± 4.9 | 53.7 ± 3.9 | 44.2 ± 5.3 | 52.1 ± 3.6 | 37.7 ± 8.2 |
| DKK1+FRZB+WNT3A | 57.8 ± 6.7 | 37.6 ± 7.2 | 54.1 ± 5.8 | 41.8 ± 9 | 51.6 ± 6.5 | 40.1 ± 9 |
| DKK1+FRZB | 53.8 ± 5.7 | 39.5 ± 6.2 | 54.3 ± 3.5 | 43.3 ± 4.6 | 53 ± 4.2 | 46.8 ± 5.2 |
| IL1β | 61.7 ± 3.6 | 49.5 ± 6.1 | 52.3 ± 6.3 | 41.9 ± 9.5 | 49 ± 6.1 | 39 ± 7.3 |
| IL1Ra+IL1β | 55 ± 3.8 | 47.2 ± 4.6 | 51.1 ± 7.2 | 44.7 ± 8.7 | 53.2 ± 5.6 | 39.7 ± 8.8 |
| IL1Ra | 55.3 ± 4.4 | 46.2 ± 7.5 | 53 ± 4.2 | 43.3 ± 6.1 | 55.4 ± 6.2 | 45.2 ± 6.9 |
| 1400W+IL1β | 59.8 ± 5.6 | 50.5 ± 7.2 | 54.5 ± 5 | 47.5 ± 5.1 | 48.8 ± 5.3 | 35.3 ± 6.9 |
| 1400W | 54.9 ± 3.3 | 44.3 ± 5.4 | 53.2 ± 4.4 | 42.1 ± 6 | 51.5 ± 5.1 | 40 ± 7.6 |

***Table S4. Ratio of unbound SOX9-mGFP per cluster, per health state of hPCs.***

|  | Healthy | | Preserved | | OA | |
| --- | --- | --- | --- | --- | --- | --- |
|  | Cluster 1 | Cluster 2 | Cluster 1 | Cluster 2 | Cluster 1 | Cluster 2 |
| Control | 0.84 ± 0.29 | 0.87 ± 0.28 | 1.25 ± 0.39 | 1.43 ± 0.35 | 1.28 ± 0.42 | 1.26 ± 0.41 |
| BMP7 | 0.86 ± 0.35 | 1.06 ± 0.3 | 1.29 ± 0.43 | 1.19 ± 0.51 | 1.09 ± 0.45 | 1.11 ± 0.42 |
| GREM1+BMP7 | 1.28 ± 0.37 | 1.18 ± 0.36 | 1.24 ± 0.32 | 1.2 ± 0.46 | 1.22 ± 0.43 | 1.04 ± 0.53 |
| GREM1 | 1.3 ± 0.43 | 1.16 ± 0.37 | 1.33 ± 0.37 | 1.38 ± 0.39 | 1.27 ± 0.39 | 1.35 ± 0.43 |
| WNT3A | 1 ± 0.32 | 1 ± 0.26 | 1.31 ± 0.38 | 1.28 ± 0.38 | 1.19 ± 0.37 | 1.23 ± 0.45 |
| DKK1+FRZB+WNT3A | 0.93 ± 0.37 | 1.04 ± 0.28 | 1.18 ± 0.4 | 1.31 ± 0.41 | 1.23 ± 0.39 | 1.13 ± 0.33 |
| DKK1+FRZB | 1.26 ± 0.35 | 1.32 ± 0.42 | 1.34 ± 0.44 | 1.25 ± 0.42 | 1.01 ± 0.34 | 1.2 ± 0.43 |
| IL1β | 0.93 ± 0.36 | 1.03 ± 0.38 | 1.32 ± 0.39 | 1.36 ± 0.35 | 1.22 ± 0.46 | 1.27 ± 0.43 |
| IL1Ra+IL1β | 1.27 ± 0.33 | 1.25 ± 0.32 | 1.28 ± 0.38 | 1.38 ± 0.38 | 1.17 ± 0.38 | 1.23 ± 0.42 |
| IL1Ra | 1.14 ± 0.36 | 1.2 ± 0.4 | 1.19 ± 0.37 | 1.29 ± 0.38 | 1.37 ± 0.45 | 1.07 ± 0.34 |
| 1400W+IL1β | 0.95 ± 0.44 | 1.05 ± 0.44 | 1.46 ± 0.39 | 1.46 ± 0.48 | 1.25 ± 0.48 | 1.21 ± 0.38 |
| 1400W | 1.28 ± 0.49 | 1.34 ± 0.46 | 1.43 ± 0.38 | 1.48 ± 0.35 | 1.14 ± 0.36 | 1.2 ± 0.36 |

***Table S5.* Recovery half-time of unbound *SOX9-mGFP (A_1_) bound to DNA per cluster, per health state of hPCs.***

|  | Healthy | | Preserved | | OA | |
| --- | --- | --- | --- | --- | --- | --- |
|  | Cluster 1 | Cluster2 | Cluster 1 | Cluster 2 | Cluster 1 | Cluster2 |
| Control | 1.81 ± 0.39 | 2 ± 0.6 | 1.5 ± 0.39 | 1.42 ± 0.39 | 1.5 ± 0.41 | 1.59 ± 0.44 |
| BMP7 | 1.88 ± 0.44 | 1.74 ± 0.4 | 1.57 ± 0.43 | 1.89 ± 0.63 | 1.99 ± 0.64 | 1.68 ± 0.49 |
| GREM1+BMP7 | 1.72 ± 0.54 | 1.55 ± 0.44 | 1.41 ± 0.36 | 1.76 ± 0.51 | 1.63 ± 0.62 | 2.03 ± 0.8 |
| GREM1 | 1.47 ± 0.52 | 1.41 ± 0.18 | 1.27 ± 0.33 | 1.48 ± 0.4 | 1.48 ± 0.38 | 1.49 ± 0.47 |
| WNT3A | 1.74 ± 0.47 | 1.72 ± 0.43 | 1.49 ± 0.37 | 1.42 ± 0.48 | 1.68 ± 0.54 | 1.45 ± 0.44 |
| DKK1+FRZB+WNT3A | 1.95 ± 0.47 | 1.85 ± 0.54 | 1.67 ± 0.51 | 1.93 ± 0.59 | 1.68 ± 0.44 | 1.82 ± 0.53 |
| DKK1+FRZB | 1.6 ± 0.39 | 1.42 ± 0.42 | 1.55 ± 0.43 | 1.54 ± 0.39 | 1.98 ± 0.53 | 1.65 ± 0.47 |
| IL1β | 1.75 ± 0.36 | 1.66 ± 0.44 | 1.33 ± 0.44 | 1.29 ± 0.3 | 1.48 ± 0.49 | 1.42 ± 0.49 |
| IL1Ra+IL1β | 1.62 ± 0.33 | 1.72 ± 0.4 | 1.57 ± 0.47 | 1.47 ± 0.4 | 1.66 ± 0.4 | 1.92 ± 0.49 |
| IL1Ra | 1.91 ± 0.6 | 1.69 ± 0.49 | 1.56 ± 0.45 | 1.8 ± 0.47 | 1.7 ± 0.42 | 1.67 ± 0.4 |
| 1400W+IL1β | 1.8 ± 0.51 | 1.87 ± 0.41 | 1.24 ± 0.32 | 1.55 ± 0.5 | 1.7 ± 0.48 | 1.5 ± 0.4 |
| 1400W | 1.4 ± 0.39 | 1.48 ± 0.48 | 1.45 ± 0.4 | 1.48 ± 0.42 | 1.65 ± 0.48 | 1.65 ± 0.47 |

***Table S6.* Recovery half-time of bound *SOX9-mGFP (A_2_) bound to DNA per cluster, per health state of hPCs.***

|  | Healthy | | Preserved | | OA | |
| --- | --- | --- | --- | --- | --- | --- |
|  | Cluster 1 | Cluster 2 | Cluster 1 | Cluster 2 | Cluster 1 | Cluster 2 |
| Control | 14.37 ± 3.17 | 17.69 ± 5.37 | 13.57 ± 3.18 | 12.9 ± 4.23 | 13.37 ± 3.71 | 13.82 ± 3.41 |
| BMP7 | 14.39 ± 2.91 | 14.65 ± 3.34 | 13.19 ± 3.52 | 16.92 ± 4.47 | 17.01 ± 4.7 | 13.13 ± 2.93 |
| GREM1+BMP7 | 14.73 ± 3.91 | 12.68 ± 4.73 | 12.12 ± 4.11 | 16.31 ± 4.41 | 13.83 ± 3.91 | 16.98 ± 5.47 |
| GREM1 | 13.66 ± 3.9 | 13.95 ± 3.36 | 11.49 ± 3.39 | 13.66 ± 4.32 | 13.33 ± 3.98 | 13.81 ± 4.04 |
| WNT3A | 14.55 ± 3.87 | 16.16 ± 4.41 | 13.31 ± 3.51 | 13.17 ± 5.19 | 13.59 ± 4.27 | 12.2 ± 4.13 |
| DKK1+FRZB+WNT3A | 15.73 ± 3.44 | 13.02 ± 3.6 | 13.08 ± 3.63 | 16.28 ± 5.37 | 14.5 ± 4.33 | 15.44 ± 5.07 |
| DKK1+FRZB | 14.51 ± 4.58 | 12.22 ± 4.31 | 14.18 ± 4.14 | 12.69 ± 3.27 | 15.88 ± 4.46 | 13.14 ± 4.35 |
| IL1β | 14.54 ± 3.22 | 14.19 ± 4.22 | 12.27 ± 3.91 | 13.07 ± 4.4 | 12.36 ± 3.84 | 12.72 ± 4.83 |
| IL1Ra+IL1β | 13.33 ± 3.04 | 14.9 ± 5.23 | 13.66 ± 4.31 | 12.58 ± 2.92 | 13.3 ± 3.01 | 18.22 ± 5.35 |
| IL1Ra | 14.64 ± 4.88 | 13.76 ± 3.6 | 13.3 ± 3.86 | 15.71 ± 4.67 | 15.55 ± 4.53 | 14.27 ± 3.8 |
| 1400W+IL1β | 15.19 ± 4.19 | 15.71 ± 3.93 | 10.51 ± 3.23 | 15.3 ± 5.07 | 15.56 ± 4.63 | 13.06 ± 4.5 |
| 1400W | 10.08 ± 2.88 | 11.25 ± 4.7 | 12.81 ± 3.83 | 14.95 ± 4.11 | 13.86 ± 4 | 14.7 ± 4.21 |
